# The Impact of Adding Taxanes to Anthracyclines on Women with Breast Cancer Receiving Adjuvant Chemotherapy

**DOI:** 10.7759/cureus.7117

**Published:** 2020-02-27

**Authors:** Erina Hilaj, Alketa Ymeri, Kleva P Shpati

**Affiliations:** 1 Oncology, National Center of Continuing Education for Health Professionals, Tirana, ALB; 2 Oncology, University Hospital Center "Mother Theresa", Tirana, ALB; 3 Internal Medicine, Albanian University, Tirana, ALB; 4 Internal Medicine, University of Medicine, Tirana, ALB

**Keywords:** taxanes-based, breast cancer, chemotherapy., albania

## Abstract

Introduction

This study aimed to analyze the impact of adding taxanes to anthracycline-based regimens on women diagnosed with breast cancer and treated with adjuvant chemotherapy.

Methods

This retrospective study included 559 female breast cancer patients who underwent adjuvant chemotherapy at the University Hospital Center “Mother Teresa” in Tirana, Albania from 2005 to 2011. Three hundred fifty-nine patients received an anthracycline-based regimen, and 200 received anthracycline-plus-taxane regimens. Common anthracycline-based regimens consisted of 5-fluorouracil 600 mg/m^2^, doxorubicin 60 mg/m^2^, cyclophosphamide 600 mg/m^2^ every three weeks for six cycles. Combined taxane-anthracycline regimens were anthracycline-based regimen in the first four cycles (doxorubicin 60 mg/m^2^, cyclophosphamide 600 mg/m^2^, docetaxel 80 mg/m^2^) followed by either weekly paclitaxel or thrice-weekly docetaxel for four cycles.

Results

Overall, after a 5-year follow-up, it was found that 148 women in the taxanes-based regimen group (74%) did not experience relapse compared with 264 women in the anthracycline-based regimen group (73.5%). The relapse status was affected by hormonal status (p: <0.001) in the taxane-based regimen. In the anthracycline-based regimen patients, the relapse status was affected by hormone status and nodal involvement (p: <0.001).

Conclusion

The taxanes-plus-anthracycline regimen was slightly more effective than the anthracycline-based regimen for breast cancer patients in terms of avoiding relapse, but the difference was not statistically significant. Therefore, adding taxanes to adjuvant chemotherapy for women diagnosed with breast cancer is not beneficial for every subgroup. Hence, the future of breast cancer therapy remains chemotherapy individualized for each patient for optimal outcomes.

## Introduction

Breast cancer in Albania is the second-most frequent cancer after lung cancer. Mortality from cancer in Albania is second only to death from circulatory system diseases [1]. Treatment of breast cancer generally involves different modalities, such as surgery, radiation, and chemotherapy. Depending on the model of risk minimization, adjuvant therapy may be responsible for a 35-72% reduction in the breast cancer mortality rate [2]. Adjuvant chemotherapy is the administration of additional therapy after primary surgery to kill or inhibit micrometastasis [3]. The success of adjuvant chemotherapy in breast cancer was reported in 1976 when Bonadonna et al. published the first report on the efficacy of cyclophosphamide, methotrexate, and fluorouracil (CMF) as adjuvant treatment for node-positive breast cancer [4].

Later trials showed that a treatment similar to CMF but which substituted epirubicin for methotrexate (CEF) was even more effective in disease-free survival (DFS) and overall survival (OS) in premenopausal women with axillary node-positive breast cancer [5]. The advantage of a three-drug combination with anthracycline over CMF was confirmed in an individual patient data meta-analysis of Early Breast Cancer Trialists' Collaborative Group [6]. Altogether, 100,000 patients were included in 123 randomized trials. Standard regimens consisted of four cycles of doxorubicin hydrochloride [Adriamycin (Pfizer, New York, NY)] and cyclophosphamide (AC) and cyclophosphamide, Adriamycin, and fluorouracil (CAF) or CEF. The meta-analysis found that anthracycline-based regimens with higher cumulative dosage than standard four-cycle AC were superior to standard CMF (response rate: 0.78; p: 0.0004).

The taxane chemotherapy class of antimicrotubule anticancer agents has been the most critical addition to the chemotherapeutic armamentarium against cancer in recent decades. Paclitaxel and docetaxel have become the most used agents in adjuvant regimens of breast cancer since their approval following excellent results in treating metastatic breast cancer and many other cancer types [7]. Taxanes are preferred in adjuvant chemotherapy of breast cancer due to their pharmacokinetic profile, consistent positive results, and convenient, intermittent, and brief infusion schedule. They are not subject to cross-resistance with anthracyclines and are more active than commonly used anthracyclines [8]. Many randomized clinical trials were conducted to investigate the role of adding taxanes to anthracyclines. Some of these trials established both OS and DFS, whereas other trials did not show any advantage from adding taxanes [9-19].

In our 2016 study, the relapse rate of the taxanes-plus-anthracycline regimen was 23%, but the study was limited by its small number of cases and a lack of comparator group [20]. We conducted the present study with a larger sample of 200 women along with a traditional chemotherapy comparator group to improve the reliability of our initial findings.

The goal of this study was to analyze the impact of adding taxanes to anthracycline-based regimens on women diagnosed with breast cancer and treated with adjuvant chemotherapy in Albania.

## Materials and methods

Our study was a retrospective analysis that included 559 Albanian female breast cancer patients who underwent adjuvant chemotherapy at the University Hospital Center “Mother Teresa” in Tirana from 2005 to 2011, with a follow-up of five years. Our study included female patients younger than 70 years with a proven diagnosis of breast cancer who underwent surgery and adjuvant chemotherapy. The study excluded patients with metastatic breast cancer, patients receiving neoadjuvant or non-anthracycline-based chemotherapy, and those whose follow-up was shorter than five years.

The adjuvant chemotherapy regimens for patients in the study were anthracycline-based regimen (CAF) or an anthracycline-plus-taxane regimen (AC/T). The most common CAF regimen consisted of 5-fluorouracil 600mg/m2, doxorubicin 60mg/m2, and cyclophosphamide 600mg/m2 every three weeks for six cycles. The combined taxane-anthracycline regimen consisted of anthracycline-based regimen in the first four cycles (AC/T; doxorubicin 60 mg/m2, cyclophosphamide 600 mg/m2, docetaxel 80 mg/m2) and either weekly paclitaxel or thrice-weekly docetaxel for four cycles.

Hormone receptor assays were performed. Estrogen and progesterone receptors were considered positive if >1% of tumor cells showed expression by immunohistochemistry (IHC). Human epidermal growth factor receptor 2 (HER2)-positive patients had a score of 3+ by IHC. Most of the patients with overexpressed HER2+ received adjuvant trastuzumab.

Patients were assigned into two groups based on the type of adjuvant chemotherapy regimen received: anthracycline-based regimen and taxane-based regimen. The primary endpoint of this study was DFS, defined as the time from surgery to disease progression in the form of the local breast (including ductal carcinoma in situ) or nodal recurrence and metastatic disease.

Statistical analysis

Fisher’s exact test was used to compare the distribution of demographic and clinical characteristics of women according to their relapse status (relapse vs. no relapse at the end of the follow-up period) for each chemotherapy regimen (AC/T vs. CAF). We used the Kaplan-Meier method to assess chemotherapeutic effectiveness, and the two groups were compared with the log-rank test for significant changes. P-values less than 0.05 were considered statistically significant. IBM SPSS Statistics for Windows, Version 21.0. (IBM Corp., Armonk, NY) was used for all statistical analyses.

## Results

From January 2005 to December 2011, 559 patients fulfilling the inclusion criteria were included in this study. Overall, there were 200 women treated AC/T regimens and 359 women treated with CAF regimens as adjuvant chemotherapy. 

Table 1 presents the distribution of relapse time among women treated with AC/T and CAF. Fifty-two women (26%) experienced relapse within five years in the AC/T group, and 95 women (26.5%) experienced relapse within five years in the CAF group. A total of 148 women (74%) in the AC/T group had no relapse during the 5-year follow-up, and 264 women (73.5%) in the CAF group had no relapse during the same period.

**Table 1:** Distribution of relapse time among women treated with AC/T and CAF AC/T: anthracycline-plus-taxane adjuvant chemotherapy; CAF: anthracycline-based adjuvant chemotherapy

	AC/T		CAF	
Relapse time	n	(%)	n	(%)
<12 months	14	7	16	4
12-24 months	21	11	29	8
25-36 months	10	5	23	6
37-48 months	5	3	16	4
49-60 months	2	1	11	3
>60 months	148	74	264	73.5
Total	200	100	359	100

**Figure 1:**
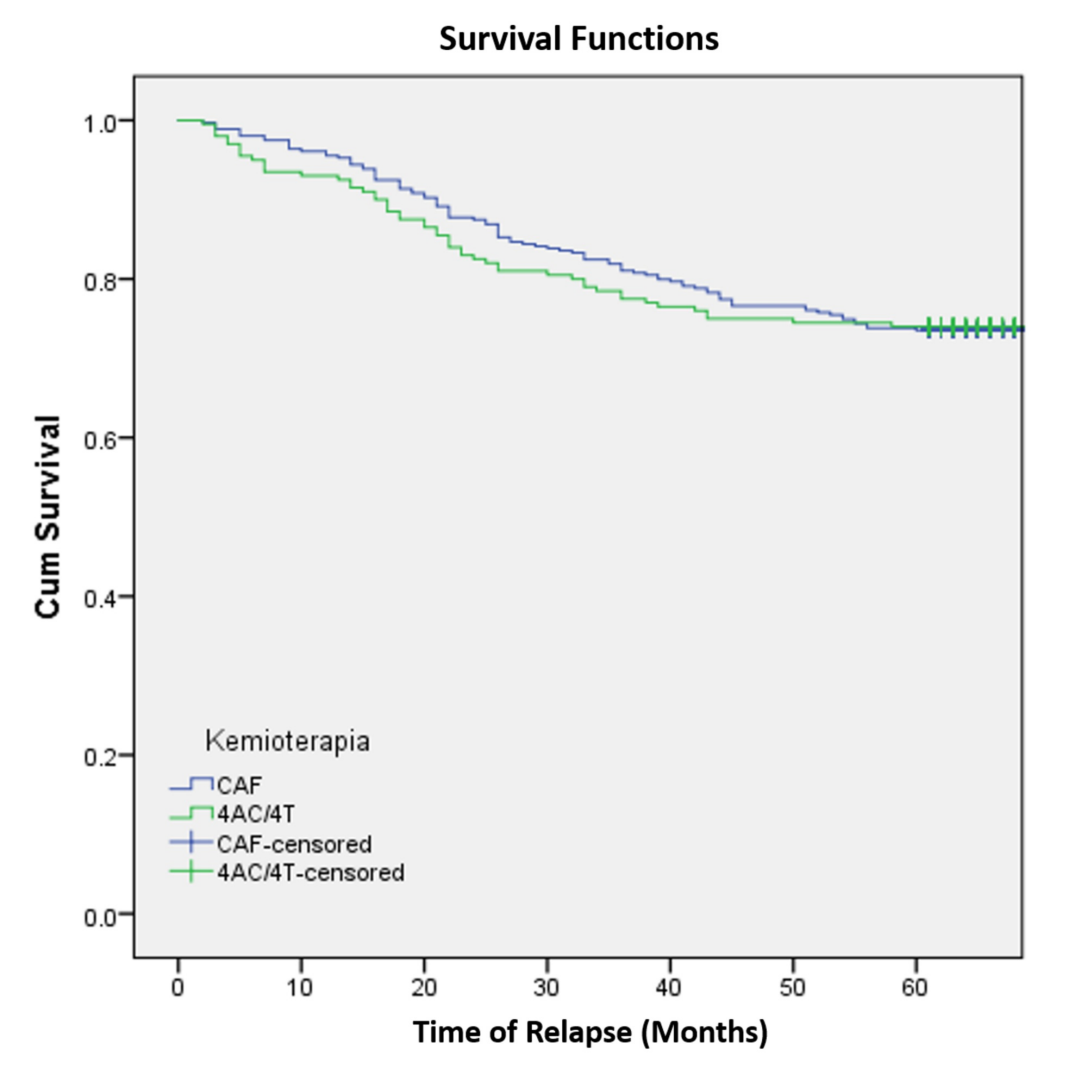
Kaplan-Meier curve for disease-free survival among patients with breast cancer based on adjuvant chemotherapy received CAF: anthracycline-based adjuvant chemotherapy; AC/T: anthracycline-plus-taxane adjuvant chemotherapy

We noted no significant difference in 5-year DFS when comparing CAF with AC/T (P: 0.943). Kaplan-Meier survival analyses are presented in Figure 1.

AC/T was a little more efficient than CAF in avoiding relapse (74 % vs. 73.5 %). However, there was no statistically significant difference between the two treatment regimens (p: 0.923).

The median DFS was higher for CAF than AC/T patients, but the total DFS was higher for the AC/T patient group (although the difference was not statistically significant; Breslow and Tarone-Ware tests, p: 0.758, p: 0.839; with a very high value of safety).

Table 2 displays the distribution of demographic and clinical characteristics, along with relapse data for each regimen. For this analysis, women were categorized into “no relapse at all” (AC/T, n = 148; CAF, n = 264) and “relapse” (AC/T n = 52; CAF, n = 95).

**Table 2:** Distribution of demographic and clinical characteristics by relapse status of Albanian woman with breast cancer treated with AC/T or CAF AC/T: anthracycline-plus-taxane adjuvant chemotherapy; CAF: anthracycline-based adjuvant chemotherapy; HER2: human epidermal growth factor receptor 2 †Fisher’s exact test

	AC/T			CAF		
Variable	Without relapse, (N = 148); n (%)	Relapse, (N = 52); n (%)	P-Value^†^	Without relapse (N = 148); n (%)	Relapse (N = 52); n (%)	P-Value^†^
Age group, years			0.454			0.227
≤35	9 (6)	3 (6)		5 (2)	3 (3)	
36-45	44 (30)	10 (19)		62 (24)	31 (33)	
46-55	61 (42)	27 (52)		130 (49)	37 (39)	
56-70	31 (21)	12 (23)		66 (25)	24 (25)	
Cancer type			0.324			0.116
Ductal	110 (77)	42 (81)		152 (59)	64 (67)	
Lobular	27 (19)	10 (19)		79 (31)	27 (28)	
Other	6 (4)	0 (0)		28 (11)	4 (4)	
Nodal involvement			0.075			0
Positive 1-3	41 (28)	10 (20)		96 (37)	21 (22)	
Positive >3	83 (57)	38 (75)		95 (36)	68 (72)	
Negative	21 (14)	3 (6)		72 (27)	6 (6)	
Hormonal status			0			0
Estrogen-progesterone negative (HER-2 unknown)	6 (4)	3 (6)		20 (8)	10(11)	
Estrogen-progesterone positive (HER-2 unknown)	16 (11)	2 (4)		64 (25)	23 (25)	
Estrogen-Progesterone positive (HER-2 negative)	48 (33)	3 (6)		112 (43)	14 (15)	
Estrogen-progesterone positive (HER-2 positive)	35 (24)	9 (17)		13 (5)	9 (10)	
Estrogen-progesterone negative (HER-2 negative)	7 (5)	22 (42)		41 (13)	12 (13)	
Estrogen-progesterone negative (HER-2 positive)	30 (21)	13 (25)		9 (3)	25 (27)	

For patients in the AC/T group, the spread of triple-negative hormone values was significantly higher among women who relapsed (42%) compared to those with no relapse (5%), but this difference was not statistically significant (p: 0.075).

For patients in the CAF group, the ratio of at least three positive nodules was significantly higher among women who relapsed (72%) compared to their non-relapsing counterparts (36%). The negative nodule ratio was significantly lower among women who relapsed (6%) compared to their non-relapsing counterparts (27%); this difference was statistically significant (p: 0.000).

## Discussion

Studies in the last few decades have consistently shown that chemotherapy produces significantly better DFS and OS [21]. Multiple components determine the necessity for patients requiring adjuvant chemotherapy. These include, but are not limited to, the tumor size, molecular subtype, histology, and tumor grade. The axillary and regional lymph node status and the tumor hormone receptor expression are also important considerations [22]. The nodal status also plays a role with any nodal involvement lowering the survival rate at five years [23].

Three randomized trials reported significant improvement in DFS by adding taxanes to the anthracyclines-based regimen in the adjuvant settings of node-positive breast cancer [14-16]. However, another three large randomized trials that included both node-negative and node-positive patients showed negative results in DFS improvement with a taxane-based regimen as adjuvant chemotherapy in breast cancer in node-negative patients [17-19].

This study supports the addition of a taxane drug as an adjuvant chemotherapy regimen in breast cancer treatment because the 5-year DFS was higher in the AC/T group, although the difference was not statistically significant. This result may have been due to the inclusion of patients regardless of their nodal status. Based on our results, identifying at least three positive nodules was associated with higher rates of relapse, which aligns with the findings reported by Miller et al. [24]. However, the prevalence of triple-negative hormonal values was much higher in AC/T treatment patients who relapsed compared to those who did not (42% vs. 5%, p: <0.001), and this change was very significant. This finding is the same as in those reported in another study where triple-negative breast cancer is considered an aggressive subtype for which the addition of taxanes to anthracycline-based regimens produced no survival benefit when compared to anthracycline-based regimens [25].

Our study had some limitations. The small number of cases, especially of those treated with the AC/T regimen, rules out broader generalization. The small number of cases obliged us to include all patients regardless of their nodal status. This limitation prohibits the study from directly illustrating the advantage of adding taxanes in breast cancer adjuvant chemotherapy. However, our results align with our previous work and indicate a potential benefit; more extensive studies that evaluate the use of taxanes independent of patient nodal status are warranted. Properly evaluating the effectiveness of adding taxanes in therapy also requires an assessment of the toxicity and side effects of an AC/T regimen, which this study could not do as we were limited by patient medical records that did not document toxicity and adverse side effects. Future prospective studies should evaluate the adverse side effects of each therapy and patient quality of life.

## Conclusions

The AC/T regimen was slightly more efficacious than the CAF regimen for breast cancer patients in terms of avoiding relapse, but the difference was not statistically significant. Therefore, we can conclude that adding taxanes to adjuvant chemotherapy for women diagnosed with breast cancer is not beneficial for every subgroup, especially those with triple-negative breast cancer. Hence, the future of breast cancer therapy remains chemotherapy individualized for each patient for optimal outcomes.
